# Socio-demographic factors influencing knowledge, attitude and practice (KAP) regarding malaria in Bangladesh

**DOI:** 10.1186/1471-2458-12-1084

**Published:** 2012-12-18

**Authors:** Kabirul Bashar, H M Al-Amin, Md Selim Reza, Muzahidul Islam, Asaduzzaman, Touhid Uddin Ahmed

**Affiliations:** 1 Laboratory of Entomology, Department of Zoology, Jahangirnagar University, Savar, Dhaka 1342, Bangladesh; 2 Laboratory of Parasitology, Centre for Communicable Disease, International Centre for Diarrhoeal Disease Research Bangladesh, Dhaka, Bangladesh; 3 Institute of Epidemiology, Disease Control & Research (IEDCR), Mohakhali, Dhaka 1212, Bangladesh

## Abstract

**Background:**

A clear understanding of the social and behavioral risk factors, and knowledge gaps, related to exposure to malaria are essential when developing guidelines and recommendations for more effective disease prevention in many malaria endemic areas of the world including Bangladesh and elsewhere in the South East Asia. To-date, the level of knowledge that human populations, residing in moderate to high malaria risk zones, have with respect to the basic pathogen transmission dynamics, risk factors for malaria or disease preventative strategies, has not been assessed in Bangladesh. The purpose of this study was to address this gap by conducting surveys of the knowledge, attitudes and practices (KAP) of people, from variable socio-demographic backgrounds, residing in selected rural malaria endemic areas in Bangladesh.

**Methods:**

The KAP survey was conducted in portions of six different malaria endemic districts in Bangladesh from July to October 2011. The survey consisted of interviewing residence of these malaria endemic districts using a structured questionnaire and interviewers also completed observational checklists at each household where people were interviewed. The study area was further divided into two zones (1 and 2) based on differences in the physical geography and level of malaria endemicity in the two zones. Data from the questionnaires and observational checklists were analysised using Statistical Package for Social Sciences 16.0 (SPSS, Inc., Chicago, IL, USA).

**Results:**

A total of 468 individuals from individual households were interviewed, and most respondents were female. Monthly incomes varied within and among the zones. It was found that 46.4% and 41% of respondents’ family had malaria within the past one year in zones 1 and 2, respectively. Nearly 86% of the respondents did not know the exact cause of malaria or the role of *Anopheles* mosquitoes in the pathogen’s transmission. Knowledge on malaria transmission and symptoms of the respondents of zones 1 and 2 were significantly (*p*<0.01) different. The majority of respondents from both zones believed that bed nets were the main protective measure against malaria, but a significant relationship was not found between the use of bed net and prevalence of malaria. A significant relationship (*p*<0.05) between level of education with malaria prevalence was found in zone 1. There was a positive correlation between the number of family members and the prevalence of malaria. Houses with walls had a strong positive association with malaria. Approximately 50% of the households of zones 1 and 2 maintained that they suffered from malaria within the last year. A significant association (*p*<0.01) between malaria and the possession of domestic animals in their houses was found in both zones. People who spent time outside in the evening were more likely to contract malaria than those who did not.

**Conclusion:**

To address the shortcomings in local knowledge about malaria, health personnel working in malaria endemic areas should be trained to give more appropriate counseling in an effort to change certain deeply entrenched traditional behaviors such as spending time outdoors in the evening, improper use of bed nets and irregular use of insecticides during sleep.

## Background

Malaria is one of the most devastating and deadly parasitic diseases in the world. It continues to be a serious public health problem in South-East Asian countries including Bangladesh [1]. It is also a social and economic burden that creates a significant barrier to economic development. For example, over a quarter of family income can be absorbed in the cost of malaria treatment, quite apart from the cost of prevention or the opportunity cost of labor lost to illness. Malaria also prevents investment and tourism into new regions, further hampering economic development [2].

**Figure 1:**
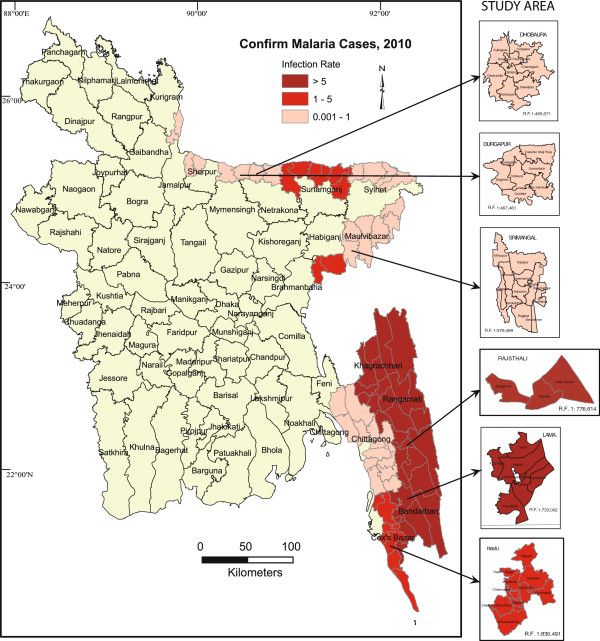
Study areas and malaria prevalence in Bangladesh.

About 33.6% of the total population in Bangladesh is at risk of malaria and the majority of cases are reported in 13 of 64 districts in the country (Figure 1) [1]. *Anopheles baimaii* Sallum and Peyton, 2005 *(dirus D), An. minimus*s.l. Theobald, 2001, *An. philippinensis* Ludlow*,* 1902*,* and *An. sundaicus* (Rodenwaldt, 1925) are considered as primary malaria vectors in Bangladesh. All four species have been reported as malaria vectors in hilly forested areas of the country while only *An. philippinensis* appears to be a vector for malaria in the lowlands or plain portion of Bangladesh [3]. Focal outbreaks of malaria are periodically reported; however, the response to these outbreaks is usually inadequate. Malaria cases are grossly under-reported due to inadequate surveillance and information dissemination systems in Bangladesh though the situation has improved in the last few years mainly because of efforts by some non-government organizations (NGOs). According to the World Health organization [1] malaria cases are presently coming down in Bangladesh though the rate of decline in morbidity could be improved. 

Current malaria control strategies in Bangladesh consist of: diagnosis and treatment of clinical cases and the promotion of use of insecticide treated nets (ITNs) or long lasting insecticide-treated nets (LLINs) [4]. The use of ITNs/LLINs is the major recommendation made by the WHO to prevent malaria [5]. To achieve its goal, the government of Bangladesh with the assistance of several NGOs have undertaken several initiatives in the malaria endemic regions of Bangladesh and these include: the use of rapid diagnostic tests, wide spread distribution of free ITNs/LLINs, retreatment of used bed nets, distribution of free drug to treat the symptoms of malaria and training for local health assistants. Training materials for health assistance where designed to allow local health assistants to manage uncomplicated malaria cases, provide appropriated advice to families regarding case reporting and proper use of ITNs/LLINs. In a developing country like Bangladesh, malaria has the potentiality to cause great morbidity and mortality and occasional outbreaks. *Plasmodium falciparum* and *P. vivax* are the key parasites in Bangladesh [6,7] although sporadic cases of *P. malariae* and *P. ovale* have also been reported recently [8,9]. *Plasmodium falciparum* is the dominant parasite and accounts for 93% of the malaria cases in the country [10]. Despite the magnitude of the problem, little documented evidence exists regarding the current knowledge, attitude and practices (KAP) of the local population as it relates to malaria and its prevention and control. Given this void, the present study was conducted to assess the current level of knowledge about malaria, its mode of transmission, sign and symptoms of disease, and prevention techniques, within the human populations residing in different malaria endemic areas of Bangladesh. The objectives of the study were to assess how different socio-demographic factors impact current patterns of knowledge and how this might influence up-take of measures to prevent or control malaria.

## Methods

### Survey areas

**Figure 2:**
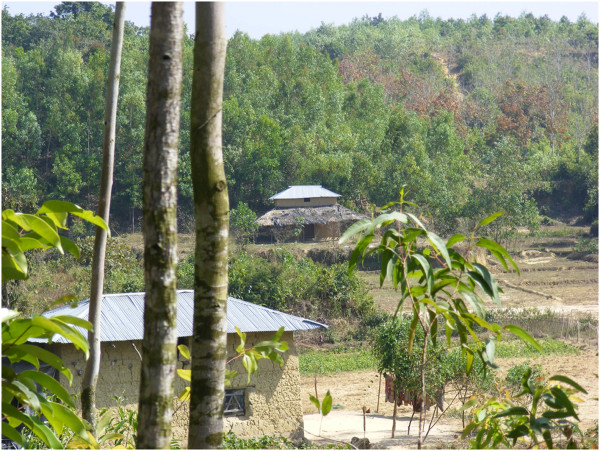
Pattern of the houses in Lama, Bandarban.

**Figure 3:**
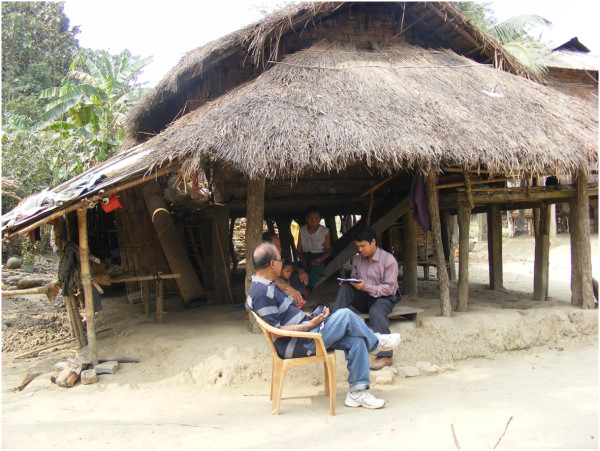
Interviews with a tribal family in Bandarban.

**Figure 4:**
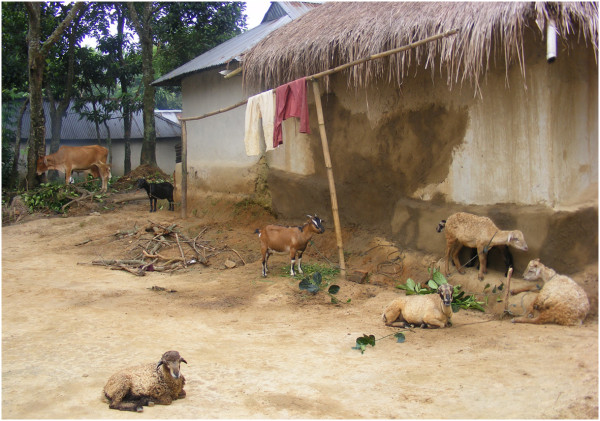
Respondent houses with domestic animal in Srimangal.

**Figure 5:**
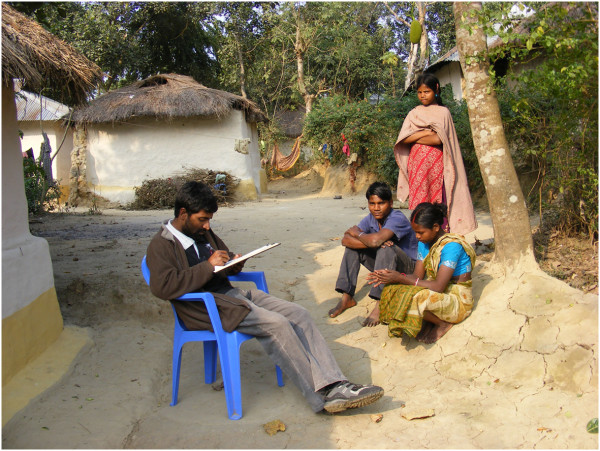
Household interviews in BTRI.

The survey was conducted in portions of six different malaria endemic districts in Bangladesh from July to October 2011. The survey consisted of interviews using a structured questionnaire and the interviewer also completed an observational checklist for each household. Households were randomly selected and sample sizes of each district were fixed on the basis of the population of the areas and the anticipated regional prevalence of malaria. The study area was divided in two zones (1 and 2) on the basis of prevalence of malaria and the physical geography of the study area. Zone 1 (4 districts) is the hilly and highly malaria endemic zone while Zone 2 (2 districts) is the lowlands or plains habitat with low malaria endemicity. Zone 1 includes Lama upazilla (sub-district) of Bandarban, Ramu of Cox’s Bazar, Rajhsthali of Rangamati, and Srimangal of Moulvibazar districts. Zone 2 consisted of Durgapur of Netrokona and Dhobaura of Mymensingh districts (Figure 1 and Table 1). The villages of Lama (Figure 2) and Ramu were inhabited by 1,206 and 1,386 people, respectively comprising both tribal and non-tribal families (Figure 3). Major parts of Lama and Ramu were vegetated by secondary forest with interspersed rubber plantations. The study area of Rajasthali had both a tribal and non-tribal population consisting of 997 people (50.3% male), living within 122 households. The survey was made in Lawachara, Magurchara, Husnabad tea estates (Figure 4) and BTRI (Figure 5) in Moulvibazar district. Almost all the laborers of these tea estates were brought from Orissa state of India during British rule. These sites consist of tea gardens, semi-evergreen coniferous forests and mixed deciduous forests [11]. Survey was also conducted in Durgapur of Netrokona and in Dhobaura of Mymenshing district where some tribal people also live. Both of these districts were within similar climatic zones with foothills, plains and undulating land and densely vegetated by secondary forest. Geographical positions of the surveyed households were recorded using handheld global positioning system (GPS-Garmin Oregon 550). ArcView GIS 3.3 and Arc GIS 9.2 software were used to map the distribution of the households where interviews were conducted. 

**Table 1:** Number of respondents and malaria cases in different malaria endemic region of Bangladesh

**Zone**	**Survey area**	**Number of respondents**	**Malaria cases**	
			**Number**	**%**
1	Bandarban	199	101	50.75
	Cox’s Bazar	40	11	27.50
	Rangamati	53	12	22.64
	Moulvibazar	72	45	62.50
	Sub-total	364	169	46.43
2	Netrokona	53	17	32.08
	Maymenshing	51	24	47.10
	Sub-total	104	41	39.42
	**Total**	**468**	**210**	**44.87**

### Sample size

In this survey, a total of 468 individuals from individual households were interviewed. The largest number of respondents (n=364) were interviewed from four malaria prone districts (zone 1). The remainder of the respondents interviewed lived within the two low malaria endemic districts bordering Meghalaya state of India (Table 1). Sample size of zone 2 was small because of low prevalence of malaria.

### Ethical approval

Ethics approval and authorization was obtained from the ethical committee of Jahangirnagar University, Bangladesh. Before data collection, each participant was clearly informed about the objectives of the study and verbal permission from the head of each household was obtained. Verbal consent was also obatin from the villagers' to capture and publish the interview photographs.

### Data collection

Households and members to be interviewed were randomly selected. The interviewers recorded the address of the interview and type of construction of the house then selected the person to be interviewed. After obtaining verbal consent, questions were asked related to: socio-demographic features of the household members, knowledge and practices regarding malaria prevention and control, treatment-seeking behavior and use of antimalarial drugs. Selected observations related to household construction were also collected using the observational checklist. In order to minimize potential biases and to improve participant understanding the questionnaire was prepared in English but translated and provided in the local language ‘Bangla’.

### Data analysis

Data analysis was done using Statistical Package for Social Sciences 16.0 (SPSS, Inc., Chicago, IL, USA). Each question was analyzed individually. Statistical relationships were sought between malaria prevalence and selected socio-demographic conditions and practices of the respondents and statistical analysis was conducted only with those families who had malaria within the last year. Results were recorded as frequencies, chi-square and *p*-values. For all purposes, *p*-value of 0.05 was considered as the level of significance.

## Results

The outcomes of the questionnaire are grouped by broad category to facilitate discussion and are as follows:

### Demographic characteristics of the respondents

Most of the respondents were female as adult male members were not within the household when the interviews were conducted (primarily during the daylight hours). We interviewed 220 (60.4%) and 34 (32.7%) female in zone 1 and zone 2, respectively (Table 1). The majority of the respondents were 25-36 years of age in zone 1 but large numbers of respondents from zone 2 were comparatively older. Approximately 78% of the respondents were married. Large portions of the rural population surveyed were illiterate. Only 17% reported completing a secondary education in zone 1 and 2. Most of the respondents (both males and females) were involved in some kinds of agricultural work (i.e., most were farmers). More than 22% of the individuals worked from their own homes and the majority of these individuals were females. From 11 to 18% of the respondents were unemployed in both the zones. Most of the respondents were Muslim (50.9%), followed by Buddhist (23.7%), Christian (20.9%) and Hindu (4.5%). Most households consisted of families with fewer than 6 members but more than a third had 6-10 family members sharing one household. Nearly 56% of the respondents from both zones were tribal ethnic community. Monthly incomes varied between the households in the two zones and in zone 1 more than 52% of the housed holds had a monthly income between $70-140 while in zone 2 more than 78% of the households earned this much monthly. A total of 46.4% and 39.4% of the respondents’ family had malaria within the past year in zone 1 and zone 2, respectively (Table 2). Almost 9.8% of the respondents reported that they had experienced about of malaria fever one month prior to the survey while 7.7% and 12% reported fever, likely due to malaria, within the last three and six months, respectively.

**Table 2:** Demographic characteristics of the respondents

	**Zone 1**		**Zone 2**		***p*-value**
**Characteristics**	**Number**	**%**	**Number**	**%**	
a) Gender					
Male	144	39.6	70	67.3	0.00
Female	220	60.4	34	32.7	
b) Age					0.05
<13	8	2.2	2	1.9	
13-24	52	14.3	15	14.4	
25-36	153	42.0	28	26.9	
37-48	79	21.7	28	26.9	
>48	72	19.8	31	29.8	
c) Educational level					
No formal education	144	39.6	26	25.0	0.01
Primary	115	31.6	53	51.0	
Secondary	62	17.0	18	17.2	
Tertiary	43	11.8	7	6.8	
d) Occupation					
Unemployed	41	11.3	19	18.3	ns
Farmer	112	30.8	33	31.7	
Daily labor	38	10.4	9	8.7	
Service	22	6.0	6	5.8	
Business	49	13.5	4	3.8	
Technician	1	0.3	2	1.9	
House work	83	22.8	24	23.1	
Student	17	4.7	7	6.7	
e) Tribe					
No	236	64.8	27	26.0	0.00
Yes	128	35.2	77	74.0	
f) No. of family member					
<6	210	57.7	65	62.5	ns
6-10	145	39.8	37	35.6	
>10	9	2.5	2	1.9	
g) Monthly income					
< 70 US$	95	26.1	14	13.5	0.00
70-140 US$	190	52.2	82	78.8	
141-280 US$	59	16.2	8	7.7	
> 280 US$	20	5.5	0	0.00	

ns = not statistically significant.

### Malaria awareness and practices

Awareness of the mechanisms of malaria transmission and measures to prevent infection was variable among the respondents from the different zones. More than 70% of the respondents knew that malaria is acquired from the bite of a mosquito; however, a reasonable proportion of people in zone 2 (13.5%) believed that malaria was contracted because of a lack of cleanliness. Only a small proportion of respondents knew that mosquitoes acquired the infection from an infected human and more respondents in zone 2 were aware of this than in zone 1 (Table 3). Increased knowledge in zone 2 may reflect the higher level of education in this zone. Nearly 86% of people did not know the exact cause of malaria and the role of anophelines in the transmission of this disease. Knowledge about the transmission cycle increased uniformly with years of schooling. The majority of the respondents in both the zones reported ‘fever with rigor’ as the most common symptom of malaria. Knowledge of modes of malaria transmission and its symptoms were significantly (*p*<0.01) different between the respondents from zones 1 and 2 (Table 3). The respondents from both zones had diverse knowledge on the preventive measures against malaria. Although it was observed that coil or aerosols were common in the households, people from both zones did not know that these would be effective to prevent malaria but rather used them to control nuisance mosquitoes in their homes. The majority of people from both zones believed that bed nets were the main protective measure against malaria. However, only a small portion of people from zone 1 (19.5%) mentioned that ITNs/LLINs can prevent malaria even though these nets were provided to them, specifically for that purpose since 2007 [10]. It was observed that information, education and communication (IEC) activities regarding the use of ITN/LLIN was poor. Not surprisingly, only a small proportion of people from either zone reported that managing mosquito breeding places was a good preventive measure. Most of the respondents of both zones reported that NGOs provided malaria treatment in their areas. None of the respondents from zone 2 sought treatment from the village doctor or drug sellers while nearly 15% of the respondents from zone 1used these sources when seeking treatment. Close to 100% of the respondents reported that they sleep inside at night but more than 50% people returned home after sunset. More than 53% of people from both zones stayed outside in the afternoon and about 5% of them spent evening hours at nearby tea stalls or market places (Table 3). 

**Table 3:** Awareness and practices of the respondents regarding malaria

**Characteristics**	**Zone 1**		**Zone 2**		***p-*value**
	**Number**	**%**	**Number**	**%**	
a) Someone gets malaria from					
Mosquito bites	282	77.5	67	64.4	0.00
Fly/insect bites	6	1.6	6	5.8	
Lack of cleanliness	14	3.8	14	13.5	
Other	62	17.0	17	16.3	
b) Malaria is transmitted by					
Bites from any mosquito	211	58.0	46	44.2	0.00
Bites from a a mosquito which fed upon a malaria patient	41	11.3	24	23.1	
Other	19	5.2	0	0.0	
Don't know	93	25.5	34	32.7	
c) Symptoms of malaria					
Fever with rigor	241	66.2	45	43.3	0.00
Intermittent fever	40	11.0	17	16.3	
Fever with sweating	16	4.4	9	8.7	
Other	6	1.6	0	0.0	
Don't know	60	16.5	33	31.7	
d) Malaria can be prevented by					
Limiting mosquito breeding	31	8.5	16	15.4	ns
Bed net	168	46.2	51	49.0	
Mosquito coil/aerosol	32	8.8	8	7.7	
ITN	71	19.5	14	13.5	
Other	7	1.9	0	0.0	
Don’t know	55	15.1	15	14.4	
e) Malaria treatment provided by					
NGO	277	76.1	94	90.4	0.01
Gov. hospital	34	9.3	10	9.6	
Village doctor	18	4.9	0	0.0	
Drug seller	35	9.6	0	0.0	
f) Enter the house previous night					
<6 pm	196	53.8	26	25.0	0.00
6 pm - 9 pm	158	43.4	76	73.1	
>9 pm	10	2.7	2	1.9	
g) Spent time last afternoon					
Inside	154	42.3	20	19.2	0.00
Outside	195	53.6	78	75.0	
Market	10	2.7	1	1.0	
Tea stall	5	1.4	5	4.8	
h) Spent most of the night time					
Inside	356	97.8	104	100.0	ns
Outside	7	1.9	0	0.0	

ns = not statistically significant.

### Association of malaria with socio demographic conditions

The highest proportion of respondents with malaria lived in Moulvibazar and Bandarban districts. Significant differences (*p*<0.01) were observed in the prevalence of malaria among the six study districts. Level of education was an important variable with respect to malaria and awareness of the disease. For example, level of education was significantly related to of prevalence of malaria (*p*<0.05) in zone 1 but not zone 2. Occupation and religious affiliation (data not shown) did not appear to be important with respect to prevalence of malaria. However, the number of family members residing within a household did significantly influence malaria prevalence. There was a positive correlation between number of family members per household and prevalence of malaria. Households with more than 6 family members had a higher prevalence of malaria. A significant relationship with average monthly income and prevalence of malaria was observed in zone 1 but not zone 2. Similarly, a strong association (*p*<0.01) was observed between the type of materials used in the partitions of the houses and prevalence of malaria in only zone 1. The majority of the household where malaria cases resided were constructed with mud walls. Likewise, more than 75% of the houses had tin roofs and partitions with floor made of mud. Almost all of the malaria cases resided in homes with tin or straw/thatched roofs and only 3 cases of malaria lived within homes built from bricks. Significant associations between the occurrence of malaria and type of roof or floor were not observed (Table 4), and this was not surprising since mosquitoes tend to rest on walls and not on floor.

**Table 4:** Association of malaria with the socio demographic conditions of the respondents

**Variables and responses**	**Malaria cases (Zone 1)**		***p*-value**	**Malaria cases (Zone 2)**		***p*-value**
	**Number**	**%**		**Number**	**%**	
a) Tribe						
No	111	47.03	ns	7	25.93	ns
Yes	58	45.31		34	44.16	
b) Educational level						
No formal education	71	42.51	0.02	10	38.46	ns
Primary	60	52.17		30	56.60	
Secondary	37	50.68		13	61.90	
Tertiary	1	11.11		0	0.00	
c) Occupation						
Unemployed	25	60.98	ns	6	31.58	ns
Farmer	51	45.54		15	45.45	
Daily labour	22	57.89		4	44.44	
Service	8	36.36		1	16.67	
Business	22	44.90		1	25.00	
House work	30	36.14		10	41.67	
Student	10	58.82		4	57.14	
d) Family member						
<6	86	40.95	0.05	20	30.77	0.03
6-10	78	53.79		19	51.35	
>10	5	55.56		2	100.00	
e) Monthly income of the family						
< 70 US$	52	54.74	0.05	6	42.86	ns
70-140 US$	75	39.47		33	40.24	
141-280 US$	27	45.76		2	25.00	
> 280 US$	15	75.00		0	0.00	
f) Roof of the house						
Straw/thatch	38	51.35	ns	17	48.57	
Tin	130	45.14		22	32.84	ns
Concrete/Cement	1	50.00		2	100.00	
g) Partition of the house						
Jute stick/bamboo	20	31.25	0.01	14	53.85	ns
Tin	3	20.00		5	25.00	
Concrete/Cement	25	53.19		4	25.00	
Mud	121	51.27		18	42.86	
h) Floor of the house						
Mud	141	47.64	ns	34	43.04	ns
Cemented	10	38.46		7	28.00	
Semi-Cemented	13	54.17		0	0	
Other	5	27.78		0	0	

ns = not statistically significant.

### Association of malaria with practices of the respondents

In total 47% and 40% of the respondents’ reported that they slept under a bed net regularly in zones 1 and 2, respectively, but still a high proportion of respondents contracted malaria. This might be explained by improper or infrequent use of bed nets. We observed that people sometimes sleep at night in open spaces (and not under a bed net) during periods of hot weather. Although most people believe that use of mosquito nets prevent malaria transmission but significant relation (*p*>0.05) between malaria cases and use of bed net was not found. Residents of zone 1 did use insecticides (especially coils) more frequently than people from zone 2. It is possible that people in zone 1 suffer from malaria more than area 2, which prompts them to used insecticides more frequently. Although a reasonable proportion of villagers from both zones used insecticides in their houses for mosquito control the effect of insecticide use on prevalence of malaria was not significant (*p*>0.05) in either zone. Interestingly, the majority of malaria cases were observed in residents living in homes with domestic animals (e.g., cattle, goat, sheep, dog, chicken and ducks). Approximately 50% of the households in zone 1 and 2 who had domestic animals suffered from malaria within the last year and this relationship was statistically significant in both zones (*p*<0.01) (Table 5). Spending a portion of the evening at outdoor meeting places was a regular practice for most of the people who acquired malaria and the majority of them returned to their houses before 9 pm. A strong statistical relationship between malaria prevalence (*p=*<0.01) and the time when people returned home was observed in both study zones (Table 5). It appears that malaria transmission is more closely associated with the places where the people spent time after sunset than other factors.

**Table 5:** Association of malaria with practices of the respondents

**Variables and responses**	**Malaria cases (Zone 1)**		***p*-value**	**Malaria cases (Zone 2)**		***p*-value**
	**Number**	**%**		**Number**	**%**	
a) Full family use of bed nets						
No	12	37.50	ns	3	37.50	ns
Yes	157	47.29		38	40.00	
b) Use insecticides						
No	98	43.75	ns	22	48.89	ns
Yes	71	50.71		19	32.20	
c) Domestic animals present						
No	51	38.35	0.02	14	29.17	0.04
Yes	118	51.53		27	48.21	
d) Usually enter the house						
<6 pm	102	52.04	0.03	15	57.69	0.05
6 pm - 9 pm	65	41.14		26	34.21	
>9 pm	2	20.00		0	0.00	
e) Usually spent time in the afternoon hours						
Inside	83	53.90	0.03	11	55.00	ns
Outside	77	39.49		28	35.90	
Market	5	50.00		0	0.00	
Tea stall	4	80.00		2	40.00	

ns = not statistically significant.

## Discussion

Based on the outcomes of this survey, almost all of the respondents were familiar with some aspects of malaria, NGO health workers were one of the main sources of information and this illustrates that NGOs are frequently in contact with villagers. The majority of the respondents were aware of the symptoms of malaria. Most were able to recognize ‘fever with rigor’ as the main symptom of malaria and again this was probably due to the educational messages delivered by NGO health workers. Recognition of the early symptoms of malaria is key to seeking early treatment. Sadly, about 25% the respondent did not know how malaria is acquired and few were aware of the modes of transmission or even the role of mosquitoes in transmission. Even when people were aware that mosquitoes were the vector of malaria, few were aware that mosquitoes became infected by feeding upon a person with malaria. This lack of knowledge was similar between the two study zones. Nearly 32% of the respondents from zone 2 were unaware about the symptoms of malaria. It may be that people from zone 2 are less familiar with the symptoms because fewer people acquire infection on an annual basis.

Economics also played a role in malaria in this study. For example, poor families had a higher prevalence of malaria and this is likely influenced by construction materials used in their homes. As has been reported by others [12], in malaria endemic areas the risk of infection is higher in poorly built houses than in well-built ones. In southern Sri Lanka, the risk of malaria was reported to be 2.5-fold higher between residents of poorly constructed houses compared to people living in houses of good construction type [13]. A similar situation between malaria prevalence and housing types were in zone 1. Malaria prevalence was also linked to family size such that large families sharing a single household tended to have a higher prevalence of malaria. This relationship may be related to the fact that large family households contain people who are generally poor, illiterate and living in poorly constructed houses. In addition, many of these individuals spent time outdoors in the late afternoon or early evening and did not sleep under a bed net.

With respect to knowledge about malaria prevention, many people were unaware of the most effective prevention methods. The lack of knowledge likely stems from a lack of IEC (Information, Education and Communication) activities in the study area.

Although NGOs were supposed to distribute ITNs to every family [14] of the study area some respondents claimed that they had yet to receive any form of bed net (ITNS or LLINS). Many respondents mentioned that a household would receive a single net but this was not sufficient for all of their family members. Indeed, the current malaria control strategy in Bangladesh relies mainly on the use of ITNs/LLINs. For several years, this strategy has benefited from a mass campaign promotion [14]. Before the introduction of treated nets, Bangladesh reported as about 50,000 confirmed malaria cases and 450 malaria deaths annually [14]. After the distribution of ITNs/LLINs, mortality came down from 450 to as low as 37 in the year 2010 [10,14]. Interestingly, in the present study, we did not find a significant effect of bed nets to reduce malaria in areas where ITNs/LLINs were distributed. In contrast, some African researcher reported that rates of malaria transmission and subsequent morbidity were lowered to 50% when ITNs/LLINs were widely distributed [15-17]. It may be that the malaria vectors in Bangladesh have developed resistance to the insecticides used in the treated nets. Unpublished reports of DG health of Bangladesh that as few as 20% of malaria vector were killed in recently performed bioassays (Personal communication with Dr. Chowdhury). Antonio-Nkondjio reported that *An. gambiae* populations in Cameroon have developed resistance to Deltamethrin used in treated nets [18]. A more thorough assessment of the susceptibility of mosquito vectors in Bangladesh to the active ingredients in ITNs/LLINs seems warranted Alternatively, there may be other factors that are reducing the efficacy of the nets including: rapid loss of insecticidal quality of the treated nets due to accumulation of dust on the nets, heat effects from cooking in the same room where the nets are stored or failure to properly wash the nets. These specific issues need additional attention in the malaria endemic area of Bangladesh.

A strong association between malaria prevalence and places where people spent time after sunset was observed in study zone 1. Some malaria vectors in Bangladesh prefer to bite mainly outdoors [19] in the early part of night. Bashar et al. [19] reported higher HBI (Human Blood index) in outdoor-collected *Anopheles* mosquitoes than ones collected in-doors. Thus people who spent time outside have more chance to get malaria [19], partly because people may be bitten more frequently outdoors. The location of acquisition of malaria may be a point that needs to be emphasized in future educational materials as other researchers in Bangladesh [20] have also noted that malaria risk may occur outdoors as well as indoors.

It has been well established that if mosquito blood meals can be diverted to wild or domestic animals, which are not the reservoir host of malaria, then the number of malaria cases can be reduced in a given area [21]. This is counter to what we observed in this study, where persons who kept domestic animals, had more malaria. The present observation supports the results of Idress and Jan [22]. They reported that malaria parasite rate were greater among the children of families who kept cattle than among those who did not. In some recent studies, *Plasmodium* parasites were detected serologically in some zoophilic *Anopheles* mosquitoes in the same areas where the current study was performed [23-25]. Thus some of these zoophilic mosquito species may feed upon humans and potentially could transmit malaria [19,25]. Further research is needed to clarify the vector status of some of these zoophilic mosquito species.

## Conclusion

In the present survey, it was clear that the majority of the respondents did not have adequate knowledge on how malaria is transmitted. Similarly, many people don’t know the preventive measures to take to minimize potential exposure to malaria. This lack of knowledge is likely due to inadequate coverage with IEC activities.

Based on the results of this study, enhanced health education following the IEC system may be effective in improving the knowledge of residents within these rural communities. Health personnel working under health department and NGOs may need to be retrained in order to change certain deeply entrenched activities that put people at risk of exposure to malaria infected mosquitoes. For example, residents should be counseled to avoid spending time outdoors at peak risk periods, on the proper use of bed nets and how and when to best use insecticides in the home. Similarly, people should be notified of the potential risks of malaria (and how to minimize them) that arise when one stays outdoors after sunset. Effective malaria preventive methods should be affordable and readily available to the most vulnerable populations, as should access to treatment. Lastly, entomological surveillance should be continued and effectiveness of INTs/LLINs should be continuously evaluated in these highly malaria endemic areas of Bangladesh.

## Competing interests

The authors declare that they have no competing interests.

## Authors’ contributions

KB designed the study. KB, HMA, MSR, MI, A and TUA performed the survey. KB analyzes the data. KB, HMA, MSR, MI, A and TUA collaborated to write the manuscript. All authors read and approved the final draft of the manuscript.

## Pre-publication history

The pre-publication history for this paper can be accessed here:

http://www.biomedcentral.com/1471-2458/12/1084/prepub
